# Unraveling the Potential of Electroanalgesia: A Literature Review of Current Therapeutics

**DOI:** 10.7759/cureus.61122

**Published:** 2024-05-26

**Authors:** Jyothsna Reddy, Rohan Singhal, Anand P Gaikwad, Diya Patel, Priyansh Patel, Siddharth Kamal Gandhi

**Affiliations:** 1 Department of Internal Medicine, The Tamil Nadu Dr. M.G.R. (M.G.Ramachandran) Medical University, Chennai, IND; 2 Department of Internal Medicine, Atal Bihari Vajpayee Institute of Medical Sciences, New Delhi, IND; 3 Department of Internal Medicine, King Edward Memorial Hospital, Mumbai, IND; 4 Department of Internal Medicine, Gujarat Medical Education and Research Society, Sola, Ahmedabad, IND; 5 Department of Internal Medicine, Medical College Baroda, Vadodara, IND; 6 Department of Internal Medicine, Shri M.P. (Meghaji Pethraj) Shah Government Medical College, Jamnagar, IND

**Keywords:** scrambler therapy, spinal cord stimulation (scs), deep brain stimulation, electroacupuncture, transcutaneous electrical nerve stimulation

## Abstract

Neuropathic pain (NP), arising from dysfunction in the neurological system, poses a significant challenge in pain management due to its intricate origin and unpredictable response to conventional treatments. Electroanalgesia, a collection of techniques such as transcutaneous electric nerve stimulation (TENS), peripheral electrical nerve stimulation (PENS), spinal cord stimulation (SCS), deep brain stimulation (DBS), and electroacupuncture (EA), presents a potential alternative or complementary approach. This review brings together evidence from 56 studies to evaluate the effectiveness and safety of electroanalgesia in chronic NP. It discusses the mechanisms underlying NP, the indications for electroanalgesia, and the techniques utilized, emphasizing the diverse applications and potential benefits. However, despite its potential uses, electroanalgesia has its limitations, including variable effectiveness and potential adverse effects. Furthermore, the review recognizes the limitations of the methodology and the need for further research to refine treatment protocols and enhance the understanding of electroanalgesia's role in comprehensive pain management strategies.

## Introduction and background

Neuropathic pain (NP) is a form of persistent pain resulting from the impairment or dysfunction of the neurological system. The International Association for the Study of Pain (IASP) defines NP as an occurrence that develops after an initial lesion or disorder impacts the somatosensory nervous system [1]. The manifestation of this phenomenon can be attributed to a multitude of underlying factors that impact the neurons responsible for the transmission of nociceptive signals, including autoimmune diseases such as multiple sclerosis, metabolic diseases such as diabetic neuropathy, infection, shingles and the sequel, postherpetic neuralgia, stroke, trauma, and cancer [2]. The clinical manifestations associated with NP include allodynia (pain resulting from a non-painful stimulus), hyperalgesia (an amplified perception of pain caused by a painful stimulus), and paresthesia (prickling sensation, tingling, itching, and loss of sensation) [3,4].

The primary focus of NP care is mostly on addressing clinical symptoms rather than targeting underlying causal issues. The current array of treatment options encompasses both pharmacological and nonpharmacological modalities. While the pharmacological treatment protocol for NP includes tricyclic antidepressants (TCAs), serotonin-norepinephrine reuptake inhibitors (SNRIs), antiepileptic drugs, opioid analgesics, and topical treatments, the nonpharmacological approaches include a variety of interventional and physical therapies such as peripheral nerve blockades, epidural steroid injections, radiofrequency neuroablation and neurostimulation [5,6]. Pain medications may provide temporary relief from NP, but their use can also lead to undesirable side effects or the development of unhealthy dependence [7].

Electroanalgesia is a form of neuromodulation therapy that has gained attention as an alternative or adjunct approach for managing neuropathic pain. The technique encompasses a spectrum of invasiveness, ranging from noninvasive methods such as transcutaneous electrical nerve stimulation (TENS) to minimally invasive approaches such as percutaneous electrical nerve stimulation (PENS) and electroacupuncture (EA) and extremely invasive procedures such as deep brain stimulation (DBS) and spinal cord stimulation (SCS) [8]. TENS is a widely available, easily self‐administered, and popular adjunct therapy for people with chronic NP, which entails the delivery of electrical energy from an external stimulator to the peripheral nervous system through the application of conductive gel pads on the skin [9]. The analgesic effects of TENS are believed to arise from a combination of factors involving several systems at the peripheral, spinal, and supraspinal levels [10]. There is also evidence suggesting that TENS entails the activation of the pain modulation system in the body, leading to an augmentation in the release of endogenous opioids within the central nervous system (CNS). This suppressed the transmission of painful stimuli originating from the periphery [11].

As NP encompasses a wide spectrum of conditions, each with its unique characteristics and causes, electroanalgesia constitutes an integral component of a comprehensive, multimodal strategy for the management of pain. Research into the effects of electroanalgesia is essential to determine its suitability for various NP conditions, allowing for a more targeted treatment approach, comparing its effectiveness to other treatments, ensuring safety, and optimizing treatment protocols.

## Review

Methodology

PubMed Central, MEDLINE, Google Scholar, Embase, Cochrane, and PubMed databases were searched by all authors. We searched for regular keywords and medical subject headings (MeSH). The following search strategy was selected based on the MeSH vocabulary: (( "Neuralgia/complications"[Mesh] OR "Neuralgia/drug therapy"[Mesh] OR "Neuralgia/mortality"[Mesh] OR "Neuralgia/prevention and control"[Mesh] OR "Neuralgia/radiotherapy"[Mesh] OR "Neuralgia/rehabilitation"[Mesh] OR "Neuralgia/therapy"[Mesh] )) AND ( "Transcutaneous Electric Nerve Stimulation/adverse effects"[Mesh] OR "Transcutaneous Electric Nerve Stimulation/instrumentation"[Mesh] OR "Transcutaneous Electric Nerve Stimulation/methods"[Mesh] OR "Transcutaneous Electric Nerve Stimulation/mortality"[Mesh] OR "Transcutaneous Electric Nerve Stimulation/standards"[Mesh] OR "Transcutaneous Electric Nerve Stimulation/trends"[Mesh] ). A total of 670 articles were found, and a free full-text filter was applied to yield 153 papers. Animal studies and non-English-language articles were excluded from the analysis, along with duplicate publications and gray literature. Each article was carefully reviewed by all authors, and any disagreements were thoroughly discussed until a consensus was reached. A total of 56 studies were included in the review. Table 1 shows the literature search strategies and articles identified from each database.

**Table 1:** Literature search strategies and articles identified from each database PMC: PubMed Central, CT: Clinical Trials, ICTRP: International Clinical Trials Registry Platform.

Database	Search strategy	No. of articles identified
PubMed, MEDLINE, PMC	(( "Neuralgia/complications"[Mesh] OR "Neuralgia/drug therapy"[Mesh] OR "Neuralgia/mortality"[Mesh] OR "Neuralgia/prevention and control"[Mesh] OR "Neuralgia/radiotherapy"[Mesh] OR "Neuralgia/rehabilitation"[Mesh] OR "Neuralgia/therapy"[Mesh] )) AND ( "Transcutaneous Electric Nerve Stimulation/adverse effects"[Mesh] OR "Transcutaneous Electric Nerve Stimulation/instrumentation"[Mesh] OR "Transcutaneous Electric Nerve Stimulation/methods"[Mesh] OR "Transcutaneous Electric Nerve Stimulation/mortality"[Mesh] OR "Transcutaneous Electric Nerve Stimulation/standards"[Mesh] OR "Transcutaneous Electric Nerve Stimulation/trends"[Mesh] )	115
	Neuralgia AND Transcutaneous electrical nerve stimulation	493
Cochrane/Embase/CTgov/ICTRP	Neuralgia AND Transcutaneous electrical nerve stimulation	62

Discussion

The use of electrical impulses to stimulate nerves is a pain management technique that works by interfering with pain signals sent to the brain, thus reducing the perception of pain [8]. This approach encompasses several methods such as EA, ultrasound-guided acupotomy, PENS, TENS, and peripheral nerve stimulation (PNS).

In a study conducted by Gan et al., it was reported that TENS applied at acupoints decreased postoperative pain and emesis and was as effective as ondansetron for the prevention of postoperative nausea and vomiting [12]. Moreover, TENS has proven to be an effective modality for decreasing pelvic pain and improving patient satisfaction during minor procedures, such as office hysteroscopy [13]. Further, TENS has been reported to produce short-term reductions in pain and improvement in physical activity in patients with a range of chronic pain disorders. For instance, the short- and long-term advantages of EA in individuals with persistent low back pain were documented in a study by Sator-Katzenschlager et al. [14]. However, evidence suggests that repeated PENS therapy is more effective than repeated TENS therapy in relieving chronic low back pain [15]. Moreover, TENS has been proven to be an effective treatment option for multiple other conditions, such as neck pain, knee osteoarthritis, abdominal/pelvic pain, and temporomandibular disorders [16-19].

An increasing amount of research also supports the short-term advantages of EA and PENS when used in conjunction with traditional therapies to treat a range of acute and chronic pain conditions, including fibromyalgia, myofascial neck pain, sciatica, and migraine [20-23]. Electroanalgesia has been demonstrated to be effective in minimizing postoperative pain in patients who have undergone coronary artery bypass graft surgery [24].

Chronic NP and mechanism of pain

The mechanism underlying chronic NP involves several interconnected pathways and processes. NP often arises from damage to the peripheral nerves, which can result from conditions such as diabetic neuropathy, post-herpetic neuralgia, nerve compression, or traumatic injury. Peripheral nerve injury can lead to alterations in nerve function including abnormal spontaneous activity and increased sensitivity to stimuli [25]. After peripheral nerve injury, the CNS experiences an increase in pain signals, which is referred to as central sensitization. This process involves changes in the excitability and responsiveness of the neurons in the spinal cord and brain. Heightened neuronal activity can lead to exaggerated pain responses to normally innocuous stimuli, which is a characteristic feature of NP [26]. Disruption of neurotransmitter systems within the CNS contributes to the development and maintenance of NP. Imbalances in neurotransmitters, such as glutamate, gamma-aminobutyric acid (GABA), serotonin, and noradrenaline, can alter pain signaling pathways, leading to increased pain sensitivity and decreased pain inhibition [27]. NP has inflammation as a contributing factor to its development, which leads to increased neuronal activity and heightened sensitivity in the peripheral nerves. Inflammatory mediators released following nerve injury activate immune cells, sensitize nociceptive neurons, amplify pain signals, and promote ongoing pain [28].

Chronic NP is linked to maladaptive modifications in synaptic plasticity within the nervous system. Structural and functional alterations occur in neuronal circuits involved in pain processing, perpetuating aberrant pain signaling, and contributing to the persistence of pain even after the initial injury has healed [29]. Recent research has indicated that communication between the nervous and immune systems is vital for understanding NP. Immune cells infiltrate damaged nerves and release pro-inflammatory cytokines, contributing to neuroinflammation and neuronal sensitization. Additionally, glial cells within the CNS, such as microglia and astrocytes, are activated in response to nerve injury, exacerbating pain hypersensitivity [30]. NP is characterized by dysregulation of descending pain modulation pathways, which normally inhibit or attenuate pain signals. Dysfunction in endogenous pain control mechanisms, including descending inhibitory pathways originating from the brainstem, can result in ineffective pain modulation and exacerbate chronic pain states [31].

Indications of electroanalgesia

Electroanalgesia, or the use of electrical stimulation for pain management, can be indicated for various conditions and situations as shown in Table 2. Its particular use may differ based on the unique requirements of the individual patient and the specialized knowledge of healthcare professionals. Table 2 shows various indications of electroanalgesia.

**Table 2:** Indications of electroanalgesia NP: Neuropathic pain; TENS: Transcutaneous electrical nerve stimulation.

No.	Indications	Comments
1.	Acute and chronic pain management	Electroanalgesia is a useful tool for managing both acute and chronic pain, such as NP, musculoskeletal pain, postoperative pain, and pain caused by cancer [32].
2.	Psychological pain	Some studies suggest that electroanalgesia may have a role in managing psychological pain conditions such as depression and anxiety, although more research is needed in this area [32].
3.	Neurological disorders	Electroanalgesia may be indicated for managing pain associated with neurological conditions such as multiple sclerosis, spinal cord injury, and stroke [33].
4.	Musculoskeletal disorders	Conditions like osteoarthritis, rheumatoid arthritis, fibromyalgia, and myofascial pain syndrome can benefit from electroanalgesia to reduce pain and improve functional outcomes [34].
5.	Sports injuries	Athletes with sports-related injuries, such as strains, sprains, and tendonitis, may use electroanalgesia as part of their rehabilitation regimen to relieve pain and facilitate recovery [35].
6.	Labor pain	In obstetrics, electroanalgesia techniques like TENS have been used as nonpharmacological methods to manage labor pain during childbirth [36].
7.	Palliative care	Patients with terminal illnesses or advanced cancer often experience significant pain, and electroanalgesia can be used as part of palliative care to improve their quality of life [37].
8.	Dental procedures	In dentistry, electroanalgesia techniques such as electronic dental anesthesia or TENS may be used to manage pain during dental procedures or alleviate temporomandibular joint pain [38].

Different techniques of electroanalgesia have been indicated for various chronic pain conditions. The PNS is used to treat pain and disability along the radial, medial, and ulnar nerves of the upper extremities. The tibial and peroneal nerves are frequently targeted for stimulation of the lower extremities. SCS is used to treat NP, angina pectoris, failed back surgery syndrome (FBSS), complex regional pain syndrome (CRPS), and chronic pain, including back pain, for which SCS is the sole viable treatment. Therapeutic indications for motor cortex stimulation include postictal pain, deafferentation pain, phantom pain, stump pain, NP, back pain, and thalamic pain [39,40].

TENS has been used in individuals suffering from acute pain in general, in the pre-hospital setting, paramedic pain management of femur fracture, acute low back pain, chronic pain, fibromyalgia, knee osteoarthritis, musculoskeletal conditions such as rotator cuff impingement or adhesive capsulitis, pelvic disorders such as primary dysmenorrhea, palliative care, neurological conditions such as painful diabetic peripheral neuropathy (DPN), CRPS, and pain following spinal cord injury [41]. It is also used for chronic NP, cancer pain, and opioid-resistant pain. Scrambler therapy can be used in multiple settings, including hospitals, pain management clinics, and inpatient hospice units [42].

Techniques of electroanalgesia

Transcutaneous Electric Nerve Stimulation

TENS has been extensively used to relieve pain caused by a variety of medical disorders. It entails the application of low-level electrical currents through electrodes affixed to the skin using a tiny battery-operated device. Standard TENS units are a common adjunct therapy for patients with persistent NP because they are self-administered, portable, and generally available. TENS-induced analgesia is believed to be complex, involving the peripheral, spinal, and supraspinal processes [10].

According to the pain gate theory, nociceptive activity in the dorsal horn of the spinal cord is inhibited by large-diameter (Aβ) afferent fibers, which transport sensations such as vibration and touch. This leads to reduced pain perception [2]. TENS application is a viable method for producing analgesia because it stimulates peripheral neuronal regions, which in turn produces significant large-diameter afferent activity. In addition, TENS is hypothesized to have additional effects on spinal segments, including reduced inflammation-induced dorsal horn neuron sensitization, altered levels of neurotransmitters, such as glycine and GABA, which are thought to be involved in nociceptive traffic inhibition, and modulation of the activity of glial cells in the spinal cord, which surround and support neurons. The TENS application has two modes: a high-frequency mode (50-100 Hz and above) and a low-frequency mode (10 Hz or less) [43-45].

Peripheral Electrical Nerve Stimulation

PENS is a relatively noninvasive neuromodulation technique that works differently from TENS. The latter delivers electrical stimulation through the skin, whereas the former uses electrodes placed directly in the tissue. It is postulated that electrical impulses alter peripheral nerve activity, interfering with the ability of pain signals to reach the brain. This method is similar to the principle of gate control [2]. An additional option would be to use an electrical current to stimulate the release of endogenous opioids. Two distinct electrical stimulation pulse rates can be applied using a neurostimulator device. Enkephalins should be released, but not dynorphins, upon stimulation at a low pulse rate of 2 Hz, and dynorphins should be released, but not enkephalins, with stimulation at a high pulse rate of 100 Hz [46]. PENS has been demonstrated to modify the biochemistry of the local microenvironment at the molecular level by downregulating endorphins, neurotransmitters, and mediators of local inflammation [40].

Typically, PENS is implemented using an easily understandable set of directives. This method involves either percutaneous electrode implantation using a needle inserted perpendicular to the nerve course or direct exposure of the nerve such that the electrode can be placed adjacent to it or even wrapped around it. Subsequently, the electrode was fixed to prevent further migration or displacement. Local anesthesia is usually sufficient because of the brief duration of treatment and the relatively superficial surgical site [47].

Spinal Cord Stimulation

In SCS, dorsal pathways are stimulated along with occasional stimulation of lateral pathways. The gate theory explains analgesia in SCS, where the pain in the activated segment is decreased by antidromic conduction stimulation of Aβ fibers in the dorsal columns [2]. A further explanation for the effectiveness of SCS could be that it increases endorphin levels, which are mostly generated in the periaqueductal gray matter (PAG) and raphe nuclei (opioid hypothesis) [2]. In addition, SCS stimulates the diffuse noxious inhibitor control (DNIC) pathway, which originates in the subnucleus reticularis dorsalis in the reticular formation of the medulla oblongata and terminates on wide dynamic range (WDR) neurons in the spinal cord, ultimately reaching the dorsal horn of the spinal cord. Substance P (protein), calcitonin gene-related peptide (CGRP), and the GABA system are all involved in the mechanism of action [39].

Deep Brain Stimulation

When treating intractable NP with DBS, different areas of the thalamus and other subcortical structures are targeted; however, this method is coupled with stimulation of the brain's motor cortex, which also lowers pain [39]. DBS is a frequent treatment procedure used by neurosurgeons, which involves implanting electrodes to deliver electrical currents to particular subcortical regions of the brain. Owing to its minimally invasive nature, DBS is a desirable alternative in the field of neurosurgery when compared to other surgical approaches. Patients typically tolerate DBS. With regard to neurosurgical techniques, DBS is much less risky than the other options. The procedure is categorized as neuromodulation, which permits modifications and reversibility, in contrast to earlier processes that require the formation of brain lesions [48].

Electroacupuncture

The effects of EA on NP have been well studied. This modality works by blocking the sensory and affective aspects of NP, involving several peripheral, spinal, and supraspinal pathways, along with a variety of bioactive substances, such as cytokines, signal molecules, opioids, serotonin, norepinephrine, and glutamate receptors [49]. In EA, an electric current is delivered to the acupuncture points using an electrical stimulator. EA for chronic NP often entails several weeks of treatment sessions with needles left in the body for up to 30 minutes at a time [50].

Scrambler Therapy

**Figure 1:**
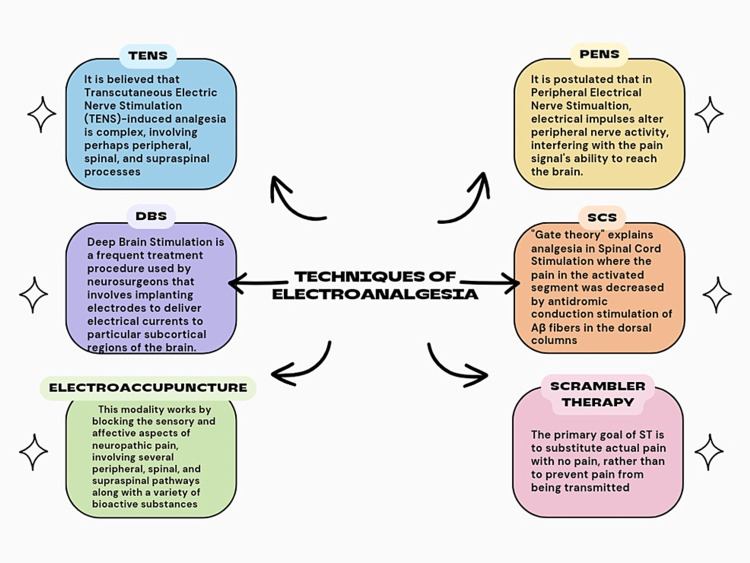
Techniques of electroanalgesia Image credit: This image was created by authors Jyothsna Reddy and Priyansh Patel.

In scrambler therapy (ST), electrodes are positioned on the healthy tissue surrounding the painful location once the patient's pain location is determined. Instead of being positioned at the actual site of pain, the electrodes were positioned close to the spot where the sensation was retained. Rather than entering the spinal cord, the dermatomal site feeds this "non-pain" confusing information into the regular neural circuit through peripheral nerves. Scrambler device sensation, which is frequently described as pleasant, vibratory, or humming, usually replaces pain when the stimulation level is adjusted based on the patient's comfort. Up to five sets of electrodes can be used to treat areas of pain [51]. The primary goal of ST is to substitute actual pain with no pain rather than to prevent pain from being transmitted. Consequently, ST can instantaneously bring the pain to zero during treatment and follow it up with a series of therapies that retrain the brain to no longer perceive the affected location as painful due to plasticity within brain networks that mediate pain perception [52]. Figure 1 illustrates the different electroanalgesia techniques.

Alternate therapies

The effectiveness of electrotherapy was assessed in conjunction with amitriptyline in the treatment of chronic painful peripheral neuropathy in individuals with type 2 diabetes. When compared to sham treatment, the degree of pain score reduction and incremental relief (above the amitriptyline effect) with electrotherapy were significantly greater. This indicates that it is a helpful supplementary modality to enhance symptom alleviation when paired with a pharmaceutical medication, such as amitriptyline [53]. In a pair of double-blind, randomized crossover experiments, Max et al. evaluated the efficacy of amitriptyline and desipramine in 38 patients and fluoxetine and placebo in 46 patients with painful diabetic neuropathy [54]. Both amitriptyline and desipramine exceeded placebo in terms of response, but the differences between fluoxetine and placebo were not statistically significant, suggesting that desipramine is a suitable alternative to amitriptyline in patients who cannot tolerate it [54].

In another study examining the efficacy of oral opioid treatments in 81 patients (58 with peripheral NP and 23 with central NP), Rowbotham et al. discovered that both groups exhibited comparable levels of improvement. Nevertheless, the authors noted that, despite a 36% average improvement in the central NP group after high-intensity therapy, a significant number of patients did not receive pain relief. In fact, 24% of the patients in the central NP group were incapable of completing the therapy because of severe and frequent side effects [55]. Bosi et al. investigated the effectiveness of the frequency rhythmic electrical modulated system (FREMS) for peripheral diabetic neuropathy in a double-blind randomized experiment. There was a significant reduction in the visual analog scale (VAS) score for both daytime and nighttime pain after all three FREMS treatment series when compared with placebo, indicating the effectiveness of FREMS on pain associated with diabetic polyneuropathy [56]. However, the VAS scores returned to the baseline value three months after the last FREMS treatment series ended, potentially indicating the need for additional treatments to sustain a clinically meaningful effect on pain over an extended period [56].

The efficacy of TENS and pulsed radiofrequency (PRF) sympathectomy was evaluated in a study by Nabi et al. in 65 patients with lower limb pain associated with peripheral diabetic neuropathy [57]. Participants were randomized to undergo TENS procedures or PRF sympathectomy, and both groups received concurrent therapy with pregabalin at doses varying from 300 to 600 mg daily. At the initial follow-up assessment, there was a noticeable decrease in the numerical rating scale (NRS) score for pain in both TENS and PRF sympathectomy patients. However, in the TENS group, NRS slowly returned to baseline values. In the PRF sympathectomy group, the NRS only slightly increased during the subsequent follow-up period but did not reach baseline levels [57]. In a 2015 study, Serry et al. examined the effects of TENS versus aerobic exercise in 60 individuals with diabetic peripheral neuropathy. Individuals who received only TENS and exercise demonstrated a statistically significant difference in pain intensity between pre-treatment and post-treatment, with a VAS improvement of 41.67% and 16.67%, respectively, suggesting that moderate-intensity TENS, administered three times a week for 30 minutes at a frequency of 14 Hz with a pulse width of 250 ms, is more effective than an aerobic exercise training program in reducing pain in patients with diabetic peripheral neuropathy [58].

Adverse effects

The adverse effects of electroanalgesia in the management of chronic NP vary depending on the modality used. TENS causes skin irritation at the site of electrode placement, uncomfortable sensations if the intensity is too high, and muscle twitching. It may also be less effective in some patients and is not recommended for patients with pacemakers or epilepsy. TENS devices have been reported to interfere with internal cardiac defibrillators (ICD), causing unintended shocks, and patients who have implanted pacemakers or defibrillators complain of dizziness and bradycardia after TENS application [59]. High atrial tracking in dual-chamber pacemakers is another possible ICD reaction to TENS [60]. TENS therapy should not be applied to the neck or head of patients with epilepsy, or in areas with bleeding, cancer, active growth plates, or during pregnancy. It is also important to exercise caution when using TENS therapy in patients with metal implants, stents, percutaneous central catheters, or drainage systems, as well as in areas near transdermal medication delivery systems [61,62].

ST is generally considered to be associated with a low risk of adverse effects. However, skin irritation can occur, and some patients may experience discomfort during the treatment sessions [42]. Risks of DBS include those associated with any surgical procedure such as infection and bleeding. Hardware-related complications can also occur, such as electrode displacement or device malfunction. Adverse effects can include cognitive changes, speech issues, and balance problems, although these are typically related to the stimulation location and can often be adjusted [63]. Potential adverse effects of SCS include surgical risks, lead migration, lead fracture, infection, pain at the implant site, and undesirable changes in stimulation, which may require additional surgery. There may also be complications involving the equipment, such as battery failure or issues with the remote control [64]. With all of these treatments, individual responses can vary greatly, and the risks must be weighed against the potential benefits for each patient. It is also important to perform these procedures with qualified healthcare professionals to minimize risks.

Limitations

Although a thorough search strategy was employed in this review, there are inherent limitations to interpreting the available information. Depending on certain criteria like "human species" and "English language" might lead to language and publishing bias, perhaps excluding important research published in languages other than English. The quality and variability of the included research may provide issues for data synthesis and the generalizability of the results. Differences in electroanalgesic procedures, patient groups, and outcome assessments in various studies could hinder the ability to make conclusive judgments on the effectiveness of electroanalgesia in chronic NP. The presence of publication bias, in which research with good results is more likely to be published, may impact the general understanding of the results. Therefore, when interpreting the results of this review, it is essential to be aware of these limitations.

## Conclusions

Understanding the multifaceted mechanisms underlying chronic NP is crucial for developing targeted therapeutic interventions aimed at restoring normal pain processing and improving the quality of life of individuals affected by this challenging condition. Effective management often involves a multimodal approach that may include pharmacological agents, physical therapies, psychological interventions, and emerging treatments, such as electroanalgesia. Electroanalgesia is a valuable multimodal approach for treating chronic NP. By targeting the function of the nervous system, these techniques offer relief and hope for individuals struggling with the debilitating effects of NP. However, similar to any treatment modality, electroanalgesia should be tailored to a patient's specific needs and considered within the context of comprehensive pain management strategies.
